# Disease Modification by Combinatorial Single Vector Gene Therapy: A Preclinical Translational Study in Epilepsy

**DOI:** 10.1016/j.omtm.2019.09.004

**Published:** 2019-09-18

**Authors:** Esbjörn Melin, Avtandil Nanobashvili, Una Avdic, Casper R. Gøtzsche, My Andersson, David P.D. Woldbye, Merab Kokaia

**Affiliations:** 1 Experimental Epilepsy Group, Epilepsy Centre, Lund University Hospital, Sölvegatan 17, 221 84 Lund, Sweden; 2 CombiGene AB, Medicon Village, Scheelevägen 2, 223 81 Lund, Sweden; 3 Laboratory of Neural Plasticity, Center for Neuroscience, University of Copenhagen, Blegdamsvej 3B, 2200 Copenhagen, Denmark

## Abstract

Gene therapy has been suggested as a plausible novel approach to achieve seizure control in patients with focal epilepsy that do not adequately respond to pharmacological treatment. We investigated the seizure-suppressant potential of combinatorial neuropeptide Y and Y2 receptor single vector gene therapy based on adeno-associated virus serotype 1 (AAV1) in rats. First, a dose-response study in the systemic kainate-induced acute seizure model was performed, whereby the 10^12^ genomic particles (gp)/mL titer of the vector was selected as an optimal concentration. Second, an efficacy study was performed in the intrahippocampal kainate chronic model of spontaneous recurrent seizures (SRSs), designed to reflect a likely clinical scenario, with magnetic resonance image (MRI)-guided focal unilateral administration of the vector in the hippocampus during the chronic stage of the disease. The efficacy study demonstrated a favorable outcome of the gene therapy, with a 31% responder rate (more than 50% reduction in SRS frequency) and 13% seizure-freedom rate, whereas no such effects were observed in the control animals. The inter-SRS and SRS cluster intervals were also significantly prolonged in the treated group compared to controls. In addition, the SRS duration was significantly reduced in the treated group but not in the controls. This study establishes the SRS-suppressant ability of the single vector combinatorial neuropeptide Y/Y2 receptor gene therapy in a clinically relevant chronic model of epilepsy.

## Introduction

Epilepsy is a common neurological disorder characterized by episodes of excessive synchronized neuronal activity, which are manifested as seizures. It is estimated that approximately 1% of the population suffers from epilepsy.^1^ Current anti-epilepsy drugs (AEDs) symptomatically suppress seizures, but they affect the whole brain and are often associated with considerable adverse effects.^2^, ^3^ Moreover, about one third of the patients do not respond adequately to the treatment.^4^, ^5^ The majority (approximately 2/3) of the pharmacoresistant cases have focal epilepsy.^5^ Surgery is an effective alternative for drug-resistant patients,^6^ but it is applicable only for the minority.^7^ Considering the severity and the high societal cost of the disease,^8^, ^9^ the development of new therapeutic strategies is highly warranted.

Reasons for the lack of progress in developing new anti-epilepsy therapies have been proposed to be the insufficiently translational design of preclinical studies as well as the challenges related to clinical translation itself.^10^, ^11^ Thus, enhancing the translational value of preclinical studies is suggested to be instrumental for the successful development of novel anti-epilepsy therapies.^11^ To achieve this goal, two main factors must be carefully considered: (1) the choice of model and (2) study design. For screening purposes and for dose-response experiments, acute seizure models in naive animals are more suitable because of high throughput capacity and still reasonably high predictive value. However, final efficacy studies should preferably be performed in clinically relevant chronic models where the pathology is more similar to the human situation, e.g., the presence of spontaneous recurrent seizures (SRS). Moreover, the design of chronic efficacy studies should be adapted to clinically relevant criteria and circumstances, particularly for the focal delivery of drugs or gene therapy products, where individualized identification and targeting of the regions of interest (ROIs) (e.g., seizure focus) using various tools, including magnetic resonance imaging (MRI),^11^ are crucial.

Gene therapy is emerging as one of the new approaches to treat neurological disorders,^12^ and indeed there are ongoing trials aimed at treating, for example, Parkinson’s disease.^13^ In epilepsy, gene therapy is gaining support based on recent advances in overexpressing genes of interest by viral vectors that counteract seizure activity in various animal models.^14^, ^15^ Neuropeptides are among such gene products that are released during high frequency activity of neurons, e.g., during seizures.^16^, ^17^ They act mostly via G-protein-coupled metabotropic receptors, and therefore have slower kinetics with more long-lasting modulatory effects.^18^ Out of several neuropeptides that have gained interest for their potential anti-epilepsy properties,^19^ neuropeptide Y (NPY) is one of the strongest candidates.^20^, ^21^, ^22^, ^23^, ^24^, ^25^, ^26^ Apart from animal studies, NPY has been shown to decrease glutamate release from principle neurons, thereby decreasing excitatory synaptic transmission^27^ as well as counteracting seizure activity in human pharmacoresistant epileptic tissue resected from drug refractory patients (unpublished data).

In the CNS, NPY acts by interacting with a set of NPY receptors, of which the Y1, Y2, and Y5 subtypes have been shown to have implications for epilepsy.^16^, ^28^ The postsynaptically located Y1 receptor promotes seizure activity,^21^, ^29^, ^30^ while the presynaptically located Y2 receptors inhibit glutamate release and seizure development^31^, ^32^, ^33^ by inhibition of voltage-gated Ca^+^ channels.^34^, ^35^ NPY gene transfer to the hippocampus has been shown to decrease glutamate release from excitatory neurons during high frequency activation, both directly in neurons participating in the activity and by volume transmission in neurons not yet recruited. This mechanism is suggested to explain the anti-epileptic effects of NPY gene therapy.^36^

Adeno-associated viral (AAV) vector-mediated combinatorial overexpression of the Y2 receptor and NPY has been shown to alter the seizure severity and progression in kindled rats.^37^ Similarly, combined Y5 and NPY overexpression resulted in a synergistic inhibitory effect on kainic acid (KA)-induced acute status epilepticus (SE) seizures.^38^ Moreover, we have demonstrated that combined overexpression of both NPY and the Y2 receptor bilaterally in the hippocampus by two separate viral vectors suppresses SRSs in a chronic model of temporal lobe epilepsy (TLE).^39^

For clinical application, a single vector containing both the ligand and the receptor genes (NPY/Y2) is selected, since the regulatory processes favor a single vector approach. In the preceding experiments to the current study, a screening for the most effective seizure-suppressant AAV-based combinatorial NPY/Y2 vector in an acute model of seizures was performed (unpublished data). The AAV1, AAV2, and AAV8 serotypes and two transgene orders (NPY-Y2 versus Y2-NPY) were compared in terms of latency to the first motor seizure, latency to status epilepticus (SE), number of convulsive seizures during SE, and total time spent in convulsive seizures. The results of these experiments supported the choice of the vector used in the present study (AAV1-NPY/Y2), based on both its transduction efficacy and best performance in suppressing kainate-induced convulsive seizures.

To provide the first proof-of-concept for the clinically relevant use of a combinatorial AAV1-NPY/Y2 gene therapy, the *single vector* was investigated for its anti-epilepsy effect. First, a dose-response study was performed, establishing the optimal concentration of the injected viral particles for seizure suppression in an acute seizure model, followed by an efficacy study in a chronic model of TLE.

The second novel proof-of-concept was targeting the *focus of seizure generation*, that is the hippocampus, *unilaterally.* The focus of the SRS was presumably created by an unilateral intrahippocampal (i.h.) KA injection, a model of post-SE (SE) *chronic epilepsy*. Moreover, the treatment was *individualized* for each animal by targeting the viral vector injections into the hippocampus based on MRI. Finally, the data were analyzed according to the outcome measures used in clinical trials, such as *responder rate*, *seizure free rate*, and *time until Nth seizure* (inter-seizure intervals).^11^, ^40^ Taken together, this experimental setup is the first of its kind to closely resemble a conceivable clinical scenario for the first-in-man gene therapy trial in epilepsy.

## Results

### Dose-Response Study

**Figure 1:**
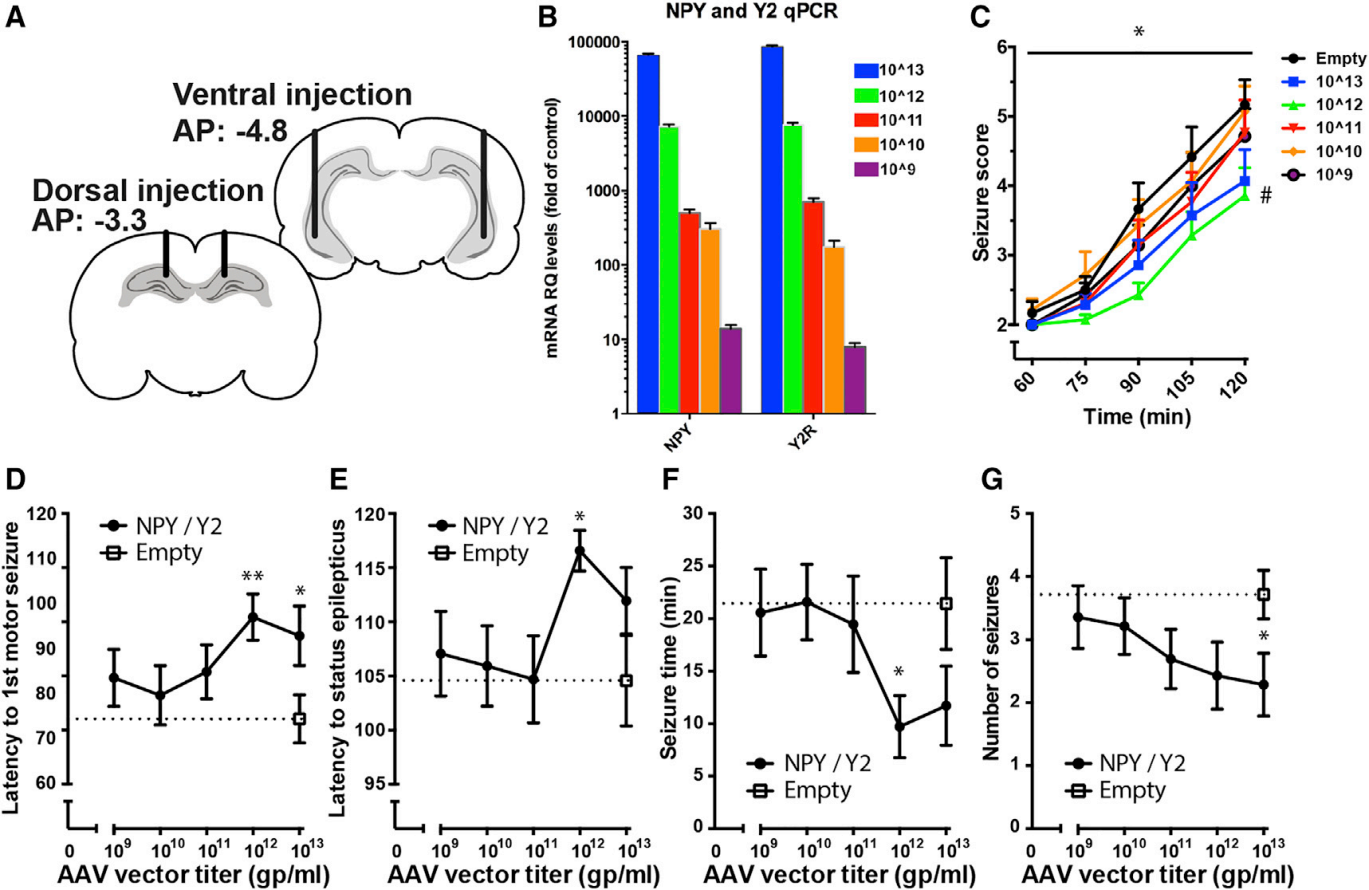
Defining the Optimal Concentration of the AAV1-NPY-Y2 (A) Coronal sections showing the locations of the bilateral viral vector delivery targeting the dorsal and ventral hippocampus. (B) The level of mRNA expressed in the transfected animals correlated to the concentration of the viral vector administrated (Pearson Correlation test, r = 0.999, p < 0.0001). (C) Seizure score as a function of time. The animals treated with 10^12^ gp/mL AAV titer exhibited a significantly lower seizure score than empty controls (significant two-way RM ANOVA, #p < 0.05 treatment effect followed by Bonferroni’s multiple comparisons test, p < 0.05). (D–G) Quantitative assessments of the seizures resulting from systemic KA injection revealed the largest seizure-suppressant effects in animals administered with 10^12^ and 10^13^ gp/mL viral vector titers. (D) The latency to the first motor seizure was prolonged in the 10^12^ and 10^13^ gp/mL group. (E) The latency to SE was prolonged in the 10^12^ gp/mL group. (F) The total time spent in seizures was shorter in the 10^12^ group. (G) The total number of seizures was fewer in the 10^13^ gp/mL group (Bonferroni adjusted Student’s t test versus control, *p < 0.05, **p < 0.01).

To optimize the NPY/Y2 gene therapy approach, a dose-response study was conducted (Figure 1). For this purpose, we utilized the systemic KA-induced SE model of acute seizures in rats injected bilaterally with viral vector, whereby latency to motor seizure development, seizure duration, and its severity were assessed according to a modified scale of Racine.^38^, ^41^

During a period of 90 min post-KA injection, the group with prior administration of 10^12^ gp/mL viral vector (NPY/Y2) showed a statistically significant reduction in seizure severity scale compared to controls (empty) (Figure 1C). Similarly, the latency to first motor seizure was longer in the two groups administered with 10^12^ and 10^13^ gp/mL NPY/Y2 compared to controls (Figure 1D). Further, only the 10^12^ gp/mL titer NPY/Y2-treated group had significantly longer latency to SE onset and spent less time in motor seizures than controls, while these parameters were not statistically different in the 10^13^ gp/mL titer group (Figures 1E and 1F). Although the number of motor seizures was not a primary outcome measure of the study, because this model is not appropriate for evaluating this parameter, the 10^13^ gp/mL group displayed a statistically significant reduction in the total number of motor seizures (Figure 1G). This trend was also seen in the 10^12^ gp/mL group.

Thus, the minimum most effective dose was achieved with 10^12^ gp/mL titer. In addition, considering that the lower most effective titer of the viral vector would be favorable in minimizing potential side effects of the treatment in future clinical trials, the 10^12^ gp/mL dose was selected for the subsequent efficacy study in a chronic model of epilepsy.

### qPCR Analysis of Transgene mRNA Expression

qPCR was performed to evaluate whether various titers of the injected viral vector were translated into respective gene expression levels in the hippocampus. Indeed, a higher administered dose/titer of the viral vector correlated with an increase in mRNA levels, both for NPY and the Y2 receptor, immediately after KA-induced seizures. The 10^12^ gp/mL vector gave an approximate 8,000-fold increase of NPY and Y2 mRNA levels compared to controls (empty), while the 10^9^ titer only resulted in a 10-fold increase (Figure 1B).

### Induction of Status Epilepticus, Development of SRSs, and Group Stratification

**Figure 2:**
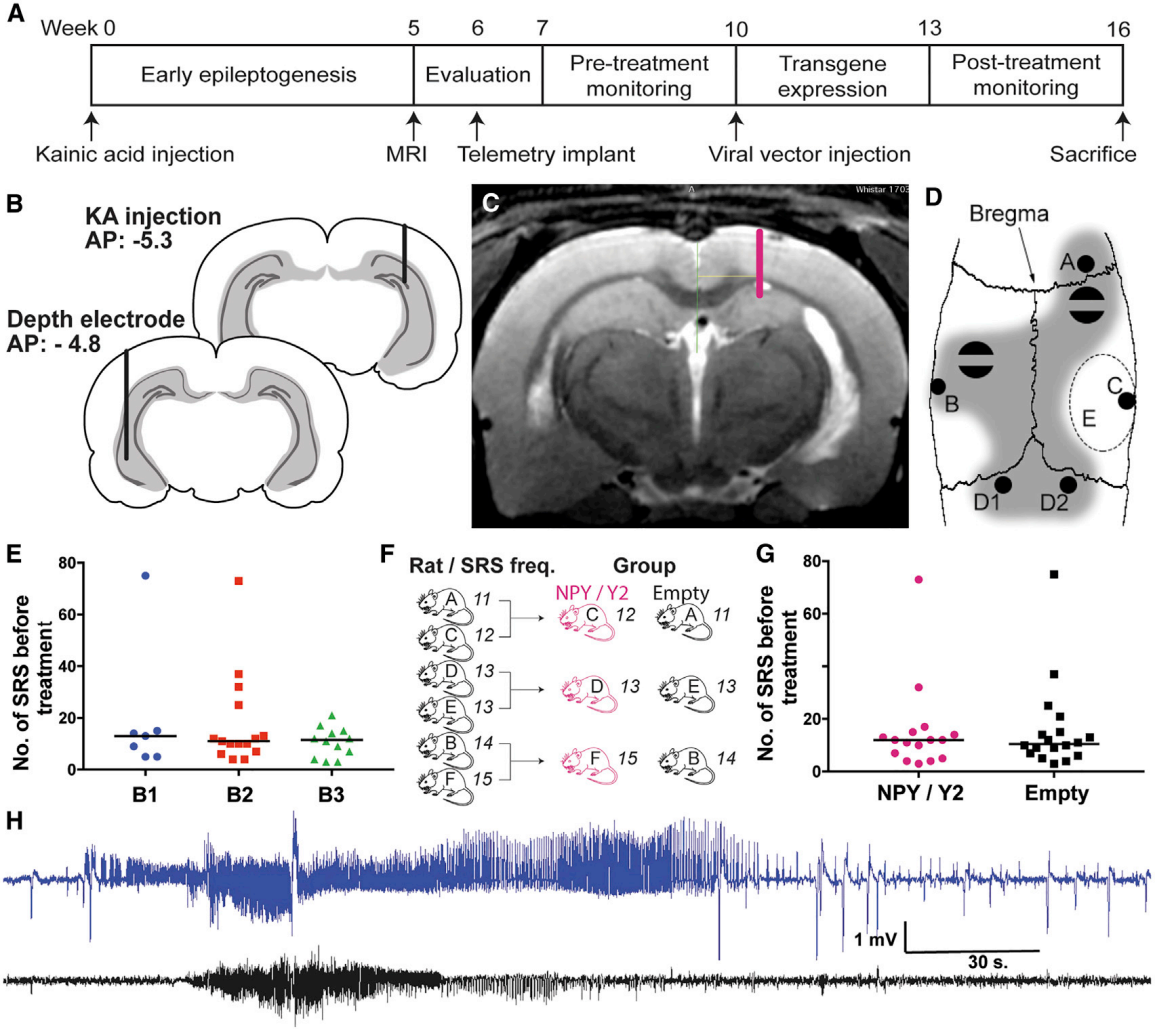
Experimental Timeline and Procedures (A) The timeline depicts the sequence of pre-treatment evaluation and monitoring, viral vector delivery, and post-treatment evaluation. (B) Two coronal sections indicating the location of the KA injection (back) and depth electrode implantation (front). (C) Typical T2 weighted MRI image of a KA injected rat. A lesion of the hippocampus is visible ipsilateral to the injection (right). The dorsal viral vector delivery location in the hippocampus for this particular animal is shown on the right side. (D) A schematic illustration of distribution of the burr holes and supporting screws on the rat skull: A, cortical electrode; B, depth electrode; C, KA injection, D1 and D2 reference electrodes; E, cement-free area reserved for viral vector delivery; shaded area, area covered with dental cement embedding two screws attached to the skull bone. (E) The number of SRS during the pre-treatment monitoring period was found not to differ between the groups, indicating equal disease severity across the three batches (p = 0.73, Kruskal-Wallis test). (F) The animals were assigned to the treatment groups with the aim of stratifying them for equal mean SRS frequency. Pairs were formed from animals with equal number of SRS, and these were then randomized into the treatment groups. (G) No difference in SRS frequency during 3 weeks of monitoring before treatment indicated a successful stratification of the groups (p = 0.93, Mann-Whitney test). (H) A typical generalized SRS found in the rats during the pre-treatment period. The hippocampal trace (blue) exhibits high amplitude fast-spiking seizure activity before the cortical manifestation of electrographic seizure becomes apparent (black trace), suggesting a focal seizure with secondary generalization.

The i.h. KA model of post-SE TLE was selected as the optimal model for creating a presumed seizure focus and investigating the SRS-suppressant effects of the focal NPY/Y2 gene therapy approach (Figure 2). This model has been shown to be resistant to carbamazepine (a common AED) treatment.^42^ A total of 84 rats were injected with KA into the right hippocampus. Due to throughput capacity reasons, the experiment was divided into three consecutive runs, and the data were then combined to increase the power of the study. For the data to be pooled, the consecutive batches had to be equal in terms of SRS frequency before treatment. Indeed, no difference was found between the three batches of animals in terms of occurrence of SRS before treatment (Figure 2E).

Out of 84 animals, 19 (23%) died as a direct consequence of SE, and 1 animal died during the KA injection procedure. Three rats were excluded from the study due to MRI-identified bilateral lesion of the hippocampus and one due to weight loss. The remaining 60 rats were implanted with electrodes for wireless video-electroencephalogram (EEG) monitoring. Two rats did not survive the surgery. After a recovery time, continuous video-EEG monitoring for 3 weeks was initiated. Out of 53 rats that were assigned for video-EEG monitoring (5 were supernumerary), 16 did not display detectable SRS (defined as highly synchronized, large amplitude oscillations in the EEG) (Figure 2H), during the first week of monitoring and were therefore excluded from further analysis. The remaining 37 epileptic rats were stratified and divided into two groups. 18 received the control (empty) vector, and 19 received the NPY/Y2 vector. Three animals were lost from the study in the NPY/Y2 group: one died during the surgery of vector administration, one was sacrificed due to excessive weight loss (humane endpoint), and one lost the electrode implant during post-treatment monitoring. The final group count resulted in 16 NPY/Y2-treated animals and 18 control (empty vector) animals.

When assigning to the treatment groups, the animals were stratified in pairs according to the individual SRS frequency to ensure an equal pre-treatment mean SRS frequency between the groups. This stratification was performed by a person who was not involved in the subsequent analysis of the study outcomes. The rats were continuously matched (yoked) based on pre-treatment SRS frequency, and then the pair was randomly assigned to the treatment or control group. Successful stratification was confirmed when no difference in SRS frequency was found between the final groups (Figures 2F and 2G). In addition, there was no overall difference in the latency to the first motor seizure after KA injection between the treatment groups (Figure S1), showing that the average epileptogenic insult was similar.

### Treatment Individualization

The MRIs were used as a basis for determining the optimal vector injection coordinates for each individual rat (Figure 2C). One injection point was aimed at the rostral hippocampus, and two were vertically aligned for the more caudal part of the dorsoventral hippocampus, all ipsilateral to the initial KA injection. The KA administration and subsequent SE resulted in a variable lesion in the right (injected) hippocampus of the animals. The viral vector injection coordinates were therefore individualized for each animal to target the remaining non-lesioned areas of the hippocampus, a presumed seizure focus, resulting in some individual variations. The mean dorsal coordinates were as follows: anterior-posterior (AP), −4.07 ± 0.26 mm; medial-lateral (ML), 2.17 ± 0.27 mm; dorsal-ventral (DV), −2.46 ± 0.16 mm. The mean ventral coordinates were as follows: AP, −5.71 ± 0.40 mm; ML, 4.86 ± 0.20 mm; DV1, −4.53 ± 0.54 mm; DV2, −6.05 ± 0.30 mm (Figure S2).

### AAV1-NPY/Y2 Treatment Decreases SRS Frequency and Total Time Spent in Seizures

The animals were monitored by a video-EEG telemetry system during 3 consecutive weeks after treatment to assess and compare SRS frequency and seizure duration to the pre-treatment period. The variability in seizure frequency during the post-treatment monitoring period was high in both groups as previously reported for the same model.^39^, ^43^ (Figure S3).

**Figure 3:**
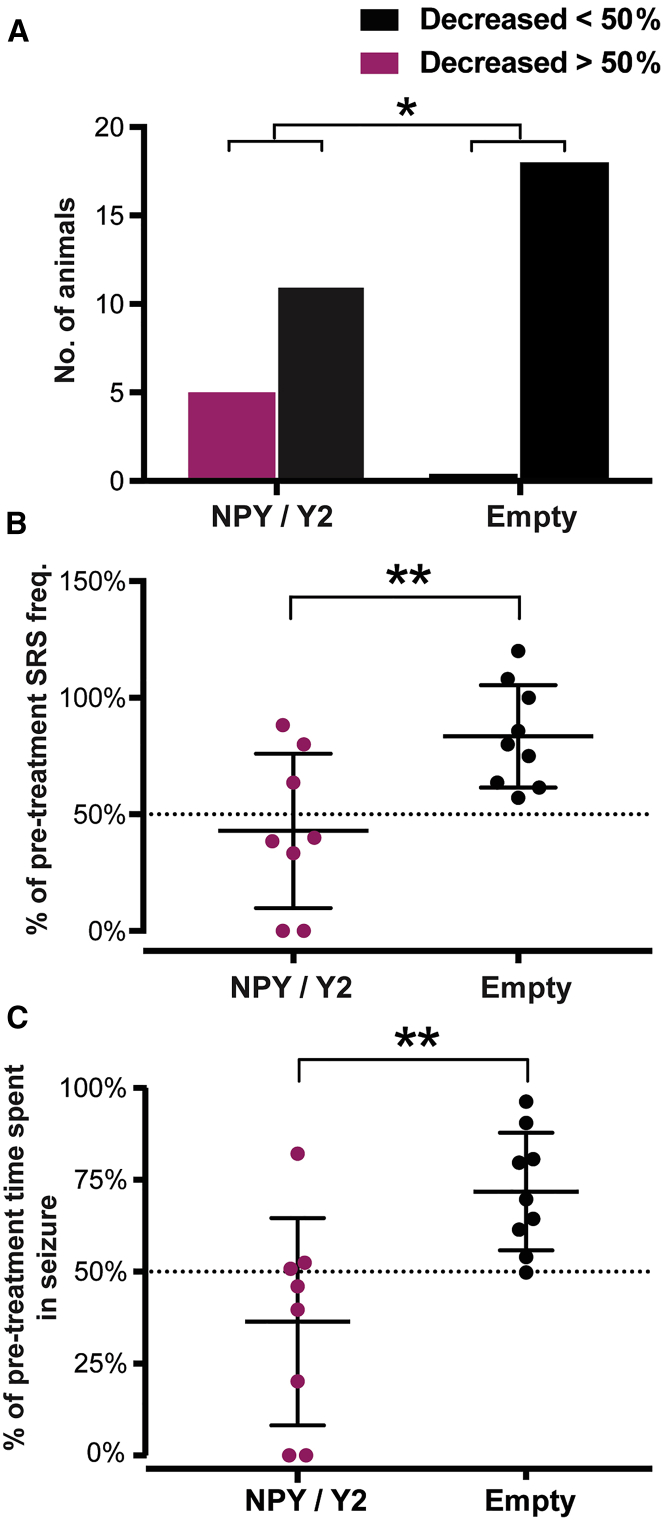
NPY/Y2-Treated Rats Exhibit Decreased SRS Frequency Compared to Controls (A) The number of animals that displayed more than a 50% reduction in SRS frequency after viral vector treatment was higher in the NPY/Y2-treated group compared to controls (p = 0.01, chi-square test). (B) The average decrease in SRS frequency in non-progressive animals (defined as a progression rate smaller than 1.21) was greater in the NPY/Y2-treated group compared to control animals. The NPY/Y2-treated group displayed an average of a 57 ± 33% reduction of SRS frequency while the control (empty) only had a 17 ± 22% reduction, significantly different form the NPY/Y2-treated group (p = 0.009, unpaired t test). (C) The non-progressive animals in the NPY/Y2-treated group also displayed a significantly greater reduction in total time spent in seizure compared to controls. The NPY/Y2-treated group had an average reduction by 64 ± 28% and the control group only 28 ± 16% (p = 0.0098, Welch’s test).

In the NPY/Y2-treated group, *the responder rate* (defined as a more than 50% reduction in seizure frequency) was 31.3%, which represents 5 of 16 animals. None of the animals in the control group (empty) exhibited a similar effect, resulting in a significant difference between the groups (Figure 3A). Two of the responding animals in the NPY/Y2-treated group were seizure free after treatment, corresponding to a *seizure-free rate* of 12.5%.

The i.h. KA-induced post-SE model is characterized by a progressive increase in SRS frequency.^39^, ^43^ The median *disease progression rate* in the control animals (calculated as a ratio of SRS frequency after treatment/before treatment) was 1.211, equivalent to a median increase in SRS frequency by approximately 20%. The animals that had less than a 20% increase, as well as a decrease in SRS frequency (*disease progression rate* < 1.211) were defined as non-progressive. In both groups, half of the animals fulfilled this criterion. However, in the NPY/Y2-treated group, the non-progressive animals exhibited a statistically significant larger average reduction (57 ± 33%) in SRS frequency than the control animals (17 ± 22%) (Figure 3B).

The SRS-suppressant effects of NPY/Y2 gene therapy were also reflected in an overall reduction in SRS duration post-treatment in the NPY/Y2 group when all animals were included in the analyses. No such difference was found in the control group (Figure S4). The effect was more pronounced in the non-progressive animals (see definition above), where the NPY/Y2-treated animals showed a significantly greater average reduction (64 ± 28%) in *total time spent in seizure* compared to control (28 ± 16%) (Figure 3C).

We then analyzed inter-SRS intervals (modified measure of *time until Nth seizure).* The occurrence of SRSs was unevenly distributed over the monitoring time (non-normal distribution) and appeared in clusters, as described previously^43^ (Figure S5). Therefore, to perform this analysis adequately, the cumulate probability curves of inter-SRS intervals (reverse measure of seizure frequency that takes into account the temporal distribution of the SRSs) were examined. Inter-SRS intervals were computed as the time passed between the seizures during 3 weeks (21 days: the total length of the monitoring session). The last SRS interval was conservatively approximated as time between the last recorded seizure and the end of the monitoring period. Resulting inter-SRS intervals were analyzed with the Kolmogorov-Smirnov test. In the pre-treatment period, no significant difference was found between the groups (Figure S6), confirming the successful stratification of the animals.

**Figure 4:**
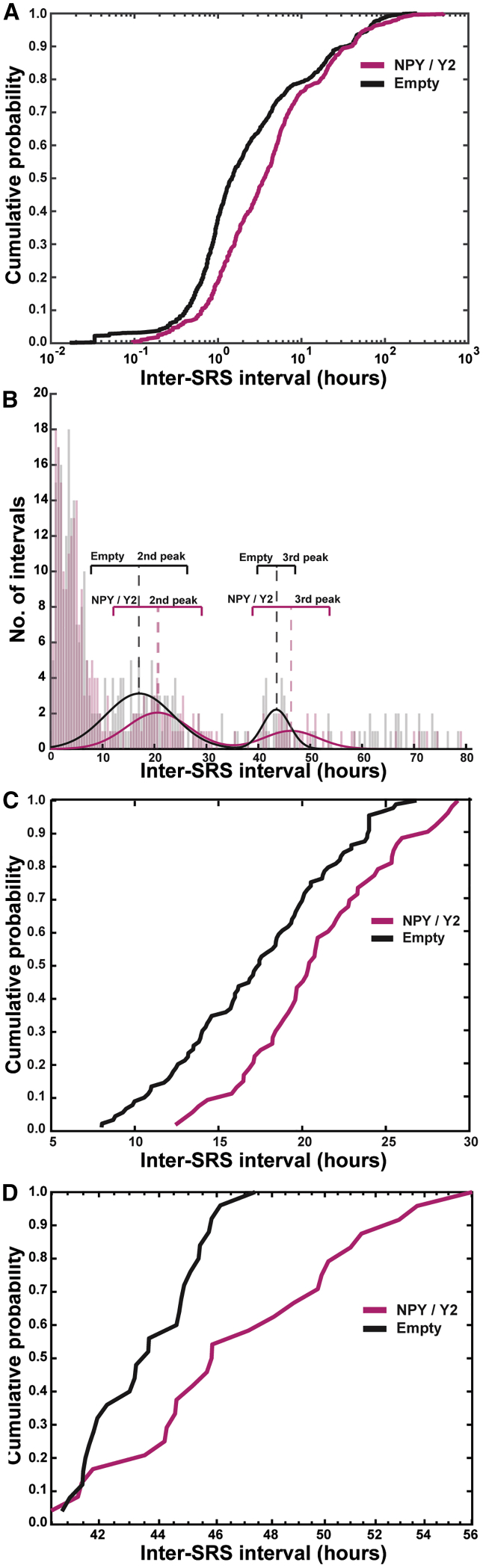
Inter-cluster and Intra-cluster Intervals Are Prolonged in Animals Treated with NPY/Y2 (A) The cumulative probability curves of the inter-SRS intervals were found to differ in animals treated with NPY/Y2 compared to controls. The significant rightward shift of the curve indicated overall longer inter-SRS intervals (p < 0.0005, Kolmogorov-Smirnov test). The most prominent difference was found between 0–10 h. (B) Multiple peaks were seen in the distribution of the inter-SRS intervals. The presence of peaks in the distribution indicated clustering of SRS, with the first peak containing intra-cluster intervals (the time between SRS within a cluster), and the second and third peak containing inter-cluster intervals (the time between SRS clusters). Automated curve fitting was used to find the center and width of the second and third peaks. (C) The second peak, related to inter-cluster intervals in the range of 5–30 h, had a statistically significant shift toward longer inter-cluster intervals in the NPY/Y2-treated animals compared to controls (p < 0.005, Kolmogorov-Smirnov test). (D) The third peak, related to inter-cluster intervals in the range of 40–60 h, contains less data points than the first and second peak. Also, this peak was statistically significantly right-shifted in the NPY/Y2 group, indicating longer inter-cluster intervals taking the small sample size into account, therefore using p < 0.05 as the level of significance (p = 0.017, Kolmogorov-Smirnov test).

In the post-treatment period, however, the same analysis revealed a statistically high significant shift of the cumulative probability curve for the NPY/Y2-treated group toward the right, indicating longer inter-SRS intervals and thereby an SRS-suppressant effect of NPY/Y2 compared to controls (empty). The Kolmogorov-Smirnov test indicated the largest differences in the lower end of the curve, suggesting that the most prominent effect was exerted on inter-SRS intervals shorter than 10 h (Figure 4A).

In addition, the cumulative probability plot revealed two plateaus at inter-SRS intervals of approximately 10 and 30 h. These plateaus reflected the SRS-free periods separating three peaks in the distribution plot (Figure 4B). The first peak contained most of the SRSs and seemed to populate the inter-SRS intervals between 0 to 10 h. Two additional lower peaks were visible with inter-SRS intervals between 10–35 and 35–55 h. The presence of peaks in the distribution plot indicated that SRSs appeared in clusters with short inter-SRS intervals (first peak), separated by seizure-free periods (longer SRS-intervals) corresponding with the second and third peaks. Hence, the inter-SRS intervals were subdivided into two categories: intra-cluster intervals (i.e., within the individual clusters) represented by the first peak (shorter than 10 h) and inter-cluster intervals represented by the following two peaks (latencies between the clusters). The inter-cluster intervals were considerably longer compared to the typical intra-cluster intervals.

In order to establish whether inter-cluster intervals were also longer in NPY/Y2-treated animals as compared to controls, a similar Kolmogorov-Smirnov test was performed on the two consecutive peaks. Gaussian curves were fitted to the second and third peak, which supplied a measure of the center and width of the distributions (Figure 4B). The underlying data were extracted and then analyzed. The Kolmogorov-Smirnov test demonstrated that, indeed, there was a statistically significant right shift in the distribution of the second and third peaks in the NPY/Y2-treated animals as compared to the controls, suggesting a prolonged latency between the SRS clusters (Figures 4C and 4D). Taken together, these data demonstrate that NPY/Y2 gene therapy treatment not only decreased the frequency of SRS, but also decreased the frequency of SRS clusters.

### Histology

**Figure 5:**
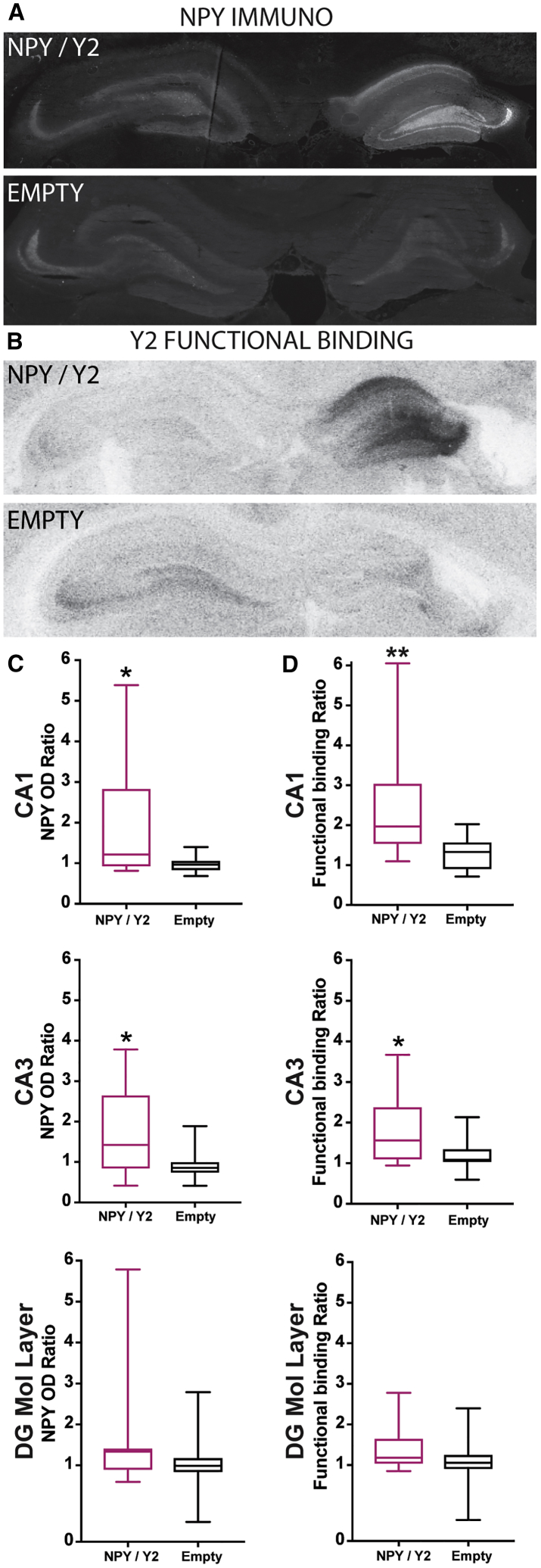
NPY and Y2 Were Significantly Increased in NPY/Y2-Treated Animals (A) A representative immunohistochemical staining of NPY in a NPY/Y2-treated animal (NPY/Y2) and a control animal (Empty). (B) Y2 receptor functional binding in a NPY/Y2-treated animal (NPY/Y2) and a control animal (Empty). (C) The dorsal CA1 and CA3 region ipsilateral to the viral vector injection had significantly higher NPY immunoreactivity compared to the contralateral side in the NPY/Y2-treated animal (p < 0.05, Mann-Whitney tests). No difference was found in the molecular layer in the dentate gyrus (p = 0.22, Mann-Whitney). (D) Y2 receptor functional binding was significantly higher ipsilateral to the viral vector injection in the dorsal CA1 (p < 0.01, Mann-Whitney) and CA3 (p < 0.05, Mann-Whitney) region. No significant difference was found in the molecular layer (p = 0.11, Mann-Whitney).

Immunohistochemical staining was performed to investigate the transgene NPY expression in the hippocampus. Qualitative assessment of immunofluorescent levels suggested a high level of NPY expression ipsilateral to the viral vector injection site in the majority of the NPY/Y2-treated animals. In particular, the dorsal CA1 and CA3 regions showed stronger NPY immunoreactivity in the NPY/Y2 group than in controls. Although transgene expression was apparent, there was a prominent variation between the individual animals as well as between the dorsal and ventral sections from the same animal. Endogenous NPY immunoreactivity was present bilaterally in the hippocampus of the control (empty) animals and contralateral to the vector injection in the NPY/Y2 group (Figure 5A).

Optical density measurements were performed to provide a semiquantitative measure of the NPY expression, and a ratio was calculated between the ipsilateral and contralateral hemispheres to the vector administration. Significantly higher expression in the vector-injected hemisphere was found in the dorsal CA1 and CA3 regions of the hippocampus in the NPY/Y2-treated group when compared to control (empty) animals. Measurements were also performed in the molecular layer of the dentate gyrus (DG), but, although showing a tendency for increase, the difference did not reach a statistical significance (Figure 5C). Similarly, Y2 receptor functional binding showed a statistically significant increase in the ipsilateral dorsal hippocampal CA1 and CA3, but not in the molecular layer of the DG of NPY/Y2 as compared to control (empty) animals (Figure 5D).

**Figure 6:**
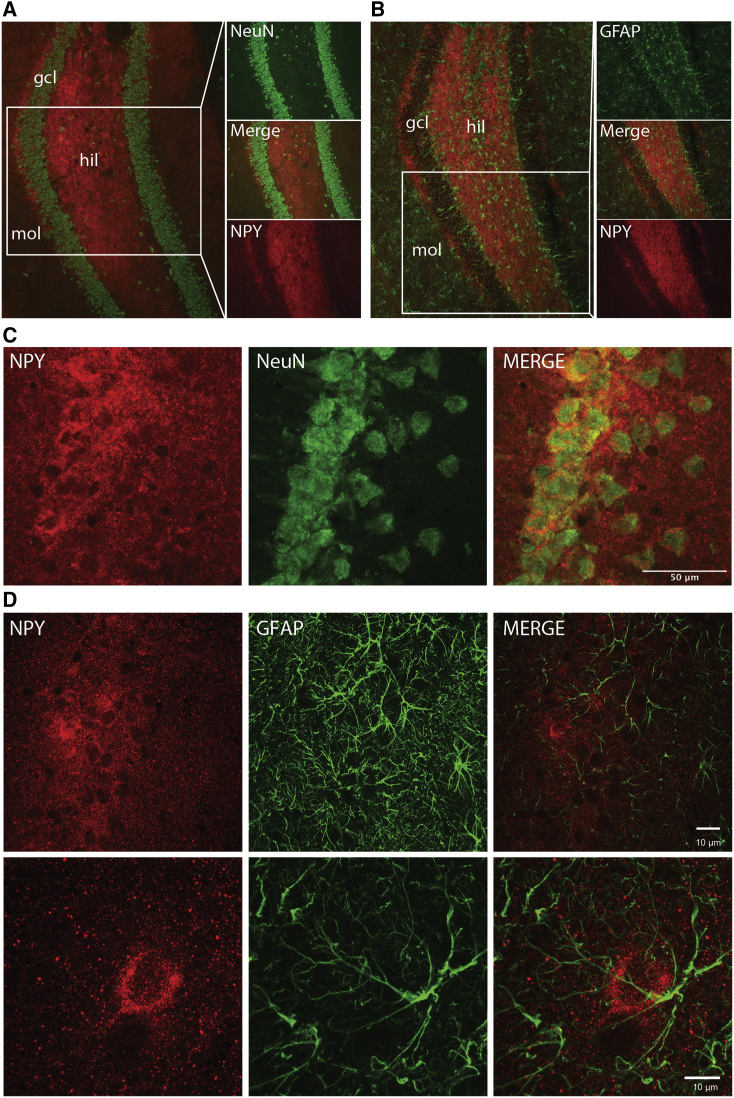
The AAV1 Serotype Displayed Neuronal Tropism (A) NPY (red) and NeuN (green, marker for neuronal cells) staining in the dorsal hippocampus dentate gyrus. (B) NPY (red) and GFAP (green, marker for astrocytes) staining in the dorsal hippocampus dentate gyrus. (C) Confocal image of CA1 indicating that NPY is present predominately in neuronal fibers. (D) Two confocal images of GFAP^+^ astrocytes (green) and NPY (red) in CA1. The glial cells do not express NPY. Scale bars in (C) 50 μm and in (D) 10 μm.

Immunohistochemical co-stainings were performed to investigate the cellular distribution of transgene NPY expression (tropism of the AAV viral vector). NPY expression was localized to neuronal fibers, with low levels of immunoreactivity in the cell soma of CA1 pyramidal neurons. Also, in the granule cell layer, NPY immunoreactivity surrounded the cell somas, indicating expression in axons and dendrites rather than in the cell body. NPY co-staining with GFAP showed that NPY was not expressed in the astrocytes (Figure 6).

## Discussion

Here we present the first proof-of-concept for effective suppression of SRS and its clustering by treatment with a *combined*, *single vector*-based NPY/Y2 gene therapy *unilaterally* in the seizure focus of a chronic model of TLE. Such a beneficial outcome in the clinically inspired preclinical study represents an important translational milestone for gene therapy in epilepsy.

### Dose-Response

In the dose-response study, we were able to reveal an effective dose (titer) of the viral vector for seizure suppression. Only concentrations of 10^12^ gp/mL and greater were effective for suppression of KA-induced seizures as compared to controls. However, the higher titer used (10^13^ gp/mL) did not exert a statistically significant effect on several parameters of the induced seizures compared to controls. A similar dose-response relationship has also been reported in AAV-based gene therapy against Parkinson’s disease.^44^ Although the extrapolation of results from the acute seizure model may not be straightforward,^12^ the fact that the seizure-inhibiting effects of 10^12^ gp/mL was, in certain parameters, superior to the 10^13^ gp/mL and to keep the delivered viral particles as low as possible for translational purposes, we choose the minimum most effective dose of 10^12^ gp/mL for the efficacy study in the chronic epilepsy model.

### Effect of Gene Therapy on SRS

The second part of the study, which addressed the efficacy of gene therapy on SRS in the chronic model of epilepsy, was designed with translation in mind, taking advantage of MRI for viral vector treatment individualization for its unilateral delivery. 16 of 53 monitored animals (≈30%) did not exhibit SRS after KA-induced SE in the pre-treatment stage and were excluded from the study prior to the treatment. Although isoflurane anesthesia has been suggested to prevent development of acquired epilepsy in post-insult models,^43^, ^45^ the rate of developing epilepsy (SRS) was similar to those reported from other studies with i.h. KA injections in awake rats (without isoflurane anesthesia).^43^, ^46^, ^47^ The lack of obvious isoflurane effect could be explained by the relatively short duration of isoflurane anesthesia in the present study (24 ± 8 min) during i.h. KA injection surgery.

The variance in SRS frequency in the KA-injected animals was relatively large and similar to previously reported data,^39^, ^43^ representing diverse disease severity. Since this variation is also the case in humans,^48^ it could be considered as a favorable aspect of the model, reiterating the human disease and contributing to the translational value of the study. Stratification of the animals before the viral vector treatment was therefore necessary to ensure equal representation of SRS frequencies in the treatment and control groups.

The SRS frequency progressively increases over time after initial KA-induced SE.^39^, ^43^ Therefore, both halting and reversing the progression of SRS frequency should be considered a beneficial disease-modifying effect. In this respect, the non-progressive NPY/Y2-treated animals exhibited a significantly larger reduction in both SRS frequency and total time spent in seizures compared to controls. The NPY/Y2-treated non-progressive animals showed more than an average 50% reduction in both SRS frequency and total time spent in seizure, which is clearly a clinically relevant positive outcome demonstrating disease modification (alleviating the disease progression^11^) exerted by the translational gene therapy approach presented here.

Seizure freedom was achieved in two animals, which is similar to our previous study,^39^ where NPY and Y2 receptor co-administration with two separate vectors resulted in one seizure-free animal. However, the seizure-free animal (1 out of 6) was the only subject that had a reduction of seizure frequency that was greater than 50%. In the present study, however, one third of the animals in the treated group exhibited more than a 50% reduction in SRS frequency, indicating a stronger therapeutic effect. It needs to be emphasized that the previous study utilized bilateral vector administration, while, in this study, the NPY/Y2 vector was injected unilaterally in the seizure focus.

Importantly, due to the synergistic effect of combinatorial AAV1-NPY/Y2 treatment, the overall responder rate and effect size in non-progressive animals were considerably higher compared to only NPY overexpression reported previously, despite the advantageous paradigm of bilateral i.h. treatment.^23^ Since the i.h. KA post-SE model used here may represent a pharmacoresistant model,^42^ the disease-modifying effects of NPY/Y2 gene therapy presented here gain even more importance as a potential treatment strategy for the drug-resistant group of TLE patients.

A rationale for choosing the i.h. KA model was to ensure a defined location of SRS focus in the hippocampus, as discussed elsewhere.^43^ However, considering broad inter-hippocampal commissural connections, it is likely that a so-called “mirror” SRS focus emerges contralateral to the KA injection.^49^ This new focus of SRS generation would escape the unilateral treatment, rendering some of the animals non-responsive, thereby compromising the outcome measures. In primates, interhemispheric hippocampal connectivity is not as pronounced as in rats, however,^50^ and therefore may not pose the same risks in rodents for the focal unilateral gene therapy treatment in humans. In our study, the electrode placement would not be able to demonstrate whether the treatment would prevent spreading of seizures originating in the mirror focus to the kainate-injected hippocampus. A study utilizing bilateral electrodes would provide more detailed information on the efficacy of the local inhibition of seizure activity. One could speculate that, if the spread of the seizures from the mirror focus to the initial one could be prevented, this may affect the progression of seizure intensification, as shown by our previous publication,^51^ and would explain the observed inhibitory effect on disease progression in the present study.

### NPY/Y2 Gene Therapy Potentially Alters Postictal Refractoriness

Continuous video-EEG monitoring^43^ revealed non-normal temporal distribution of the recorded SRSs, which were often clustered. Similar patterns of SRS clustering have been reported previously in the systemic KA,^52^ i.h. KA,^43^ and pilocarpine^53^ models of post-SE chronic epilepsy. Moreover, clustering has been shown in a rat model of perinatal hypoxia-ischemia.^54^, ^55^ Clustering of SRS is also a common feature in human epilepsies and is associated with seizure refractoriness.^56^, ^57^ Changes in ionic micro-environment, pH shifts, and an increase in levels of neuromodulators such as NPY have been attributed to elevating the seizure threshold in the postictal period, leading to an accumulation of the refractoriness that eventually terminates the cluster.^43^, ^58^ Despite these observations, SRS clustering has not been studied systematically, particularly as a therapeutic outcome.

**Figure 7:**
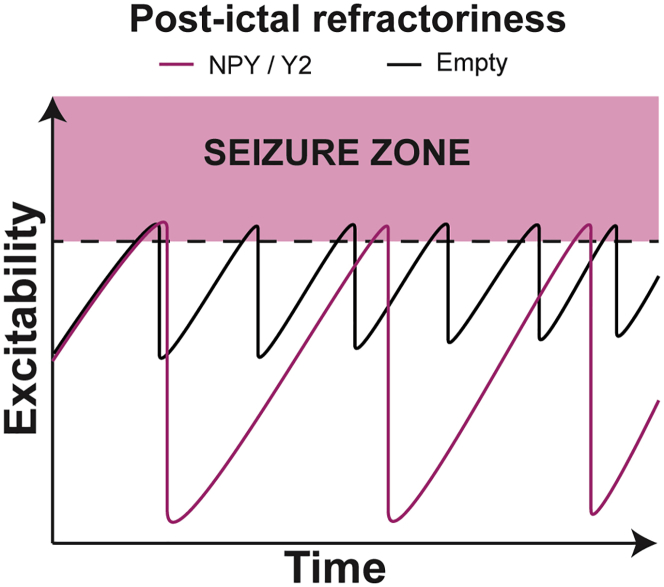
Differential NPY/Y2 Activation Dynamics in Treated Animals Might Prolong Inter-SRS Intervals and Ameliorate Disease State Seizures lead to postictal refractoriness (less excitability) that might accumulate over time, leading to termination of the SRS clusters. In control animals, the contribution from each SRS to postictal refractoriness is modest, leading to short inter-SRS intervals, both within and between clusters (black trace). We hypothesize that, in NPY/Y2-treated animals, the releasable pool of NPY is enlarged and available Y2 receptors are more abundant. Each SRS would then lead to a longer refractory period due to decreased excitability. This is reflected in longer inter-SRS intervals within and between clusters (magenta trace).

Interestingly, using cumulative probability analysis, we found that the NPY/Y2-treated group exhibited both longer intra-cluster as well as inter-cluster SRS intervals compared to the control group. One possible explanation for the beneficial changes in SRS frequency and clustering patterns may be an alteration of the kinetics of NPY synthesis, transport, release, and receptor engagement caused by NPY/Y2 overexpression. Endogenous NPY is released during high frequency activity^36^ (i.e., seizure activity), and because neuropeptide replenishment is slow,^16^, ^18^ it is possible that the releasable pool of NPY is depleted after a seizure. Indeed, levels of NPY hippocampal immunoreactivity has been shown to decrease markedly 3 h after SE.^59^ Once released, NPY interacts with its receptors both at releasing synapses and, to some extent, neighboring synapses by volume transmission.^36^ Since the Y2 receptor exhibits both low internalization and desensitization,^60^ the SRS-released NPY could lead to gradual accumulation of Y2 receptor activation that would eventually decrease neuronal circuit excitability to the level that prevents new seizures from occurring, thus terminating the SRS but also the SRS cluster. This process would be enhanced by overexpression of Y2 receptors. When no more seizures drive the release of NPY, Y2 signaling is normalized, and the process is reinitiated. Overall, combined NPY/Y2 overexpression would increase postictal refractoriness by contributing to faster NPY replenishment and accumulating Y2 activation, thereby prolonging inter-cluster and intra-cluster intervals, which translates into fewer SRSs with longer clustering intervals (Figure 7). This hypothesis needs further investigation, possibly with an NPY radioligand, and PET examination at different time intervals following a seizure.

### Transgene Expression

We observed variable transgene overexpression in the hippocampus within the AAV1-NPY/Y2-injected group. There was no direct correlation between transgene NPY expression levels as assessed by immunohistochemistry and the functional outcome. It is possible that the variability of transgene expression of NPY is due to its release from neurons caused by the proximity of the SRS to the moment of fixation of the brain, as has been previously shown after acute KA-induced seizures.^59^

The immunohistochemical analysis indicated that NPY had a predominantly neuronal expression, with no or little overlap in astrocytes. These results were in-line with what has previously been reported on serotype AAV1 expression.^61^

In a previous study, we demonstrated that combinatorial NPY/Y2 treatment did not elicit any inflammatory response different from the control vectors.^39^ This was achieved by a quantification of microglial activation by ED1 and Iba1 immunohistochemical staining, revealing no differences, and thereby providing no reason to believe that the transgene overexpression is somehow toxic to the tissue or the outcomes could be confounded by inflammatory processes.

### Conclusions

Here we demonstrate that, in a clinically relevant chronic model reflecting TLE in humans, a single AAV1-NPY/Y2 vector treatment resulted in a disease-modifying outcome on SRS frequency and duration. Further studies are required to address the safety and any effects on epilepsy co-morbidities of AAV1-NPY/Y2 treatment, and, indeed, studies are now ongoing in our lab. Taken together, this study provides an important proof-of-concept milestone toward clinical development and application of gene therapy in epilepsy, particularly for those patients that suffer from its intractable and inoperable form, opening novel avenues for gene therapy as an alternative treatment strategy in combating this severe disease.

## Material and Methods

### Animals

The dose-response study was performed by the contract research organization Pharmaseed (Israel) in compliance with The Israel Animal Welfare Act and following The Israel Board for Animal Experiments (approval no. IL-16-09-359).

The chronic experiments involving animals were performed in accordance with the Swedish Animal Welfare Agency’s guidelines, and the procedures were approved by a local ethical committee (Ethical permit no. M49-15). A total number of 84 male Wistar rats (Charles River, Sulzfeld, Germany) were used in the efficacy study. They arrived at the research facilities 1 week prior to experiment initiation weighing 200–230 g and were kept in a 12 h light/dark cycle with free access to food and water *ad libitum*. Before EEG-video monitoring commenced, the animals were housed in groups of three and thereafter singly until sacrifice. Animals were inspected daily for any signs of morbidity or mortality.

### Dose Response

The chronic efficacy study was preceded by a dose-response study with the aim of finding the optimal dose for seizure inhibition by comparing the seizure-suppressant effects of different dilutions of the AAV1-NPY/Y2 vector to the highest possible concentration of the control (empty) vector. Both the AAV1-NPY/Y2 and the AAV1-empty vector (GeneDetect, New Zealand) were driven by the synthetic CAG promoter (chicken beta-actin promoter hybridized with the CMV immediate early enhancer sequence), known to achieve strong stabile expression in human cells. The full coding sequences of NPY, prepro-NPY, and the Y2 receptor were human genes. Splicing of the ligand and receptor were ensured by the internal ribosome entry site (IRES), located between the genes. 72 Wistar rats (weighing 250–275 g at the beginning of the experiment), divided into 6 groups, were stereotaxically injected with different dilutions of the vector, ranging from 10^9^ to 10^13^ gp/mL in 10-fold increments, into the dorsal and ventral hippocampus. One group served as control, receiving an empty construct with a concentration of 10^13^ gp/mL. The viral vector administration was bilateral, infusing 1 μL at each injection point at a rate of 0.2 μL/min at the following dorsal coordinates: AP, −3.3; ML, ± 1.8; DV, −2.6; and ventral coordinates: AP, −4.8; ML, ± 5.2; DV1, −6.4; DV2, −3.8 (Figure 1A). The rats were allowed to recover for 3 weeks and then injected subcutaneously with KA (10 mg/kg) for seizure induction. Subsequent seizures were assessed and scored for 2 h according to a modified Racine scale by an operator blind to the treatment of the animals.^38^, ^41^ After 3 h, the rats were sacrificed, and the brains were saved for qPCR by freezing.

### Experimental Design and Timeline for the Chronic Efficacy Study

The experimental timeline was designed with translation to the clinic in mind. The progressive development of the disease in the model, combined with a setup of a clinically inspired examination method followed by the experimental treatment, was composed to closely mimic the clinical situation.

The experiment was performed in three overlapping batches to obtain a minimum number of 32 included animals. Rats were continuously added to two treatment groups, one receiving the treatment vector (NPY/Y2) and one receiving the control vector (empty). During the experiment, the groups were stratified to contain animals with equal disease severity in terms of mean SRS frequency (Figure 2F).

To begin with, the animals were subjected to an excitotoxic insult (i.h. KA injection) that culminated into SE, which triggers epileptogenesis, rendering the animals epileptic. Previous results have indicated that most animals experience SRS after 5 weeks.^39^ Thus, at this time point, the rats were subjected to MRI (week 5), and individual coordinates for the viral vector injection were calculated. During the following week (week 6), a telemetric device and electrodes for continuous recording of two EEG channels were implanted. The rats were allowed to recover for 1 week and were then placed under video-EEG monitoring for 3 consecutive weeks (weeks 7–9), during which time the pre-treatment SRS baseline parameters were obtained. Based on the pre-treatment data, the rats were assigned into either the NPY/Y2-treated group or the control group with the aim of stratifying the groups in terms of mean SRS frequency. The viral vector was administered during week 10 followed by 2 weeks (weeks 11–12) of recovery before video-EEG monitoring was performed. Post-treatment monitoring was continued for 3 weeks (weeks 13–15), and the rats were sacrificed thereafter (Figure 2A).

### Induction of Status Epilepticus

The i.h. injection of KA was performed as previously described.^39^ 84 rats were injected with KA into the DG of the dorsal hippocampus for them to develop SE. At the beginning of the procedure, the rat was placed in an anesthesia induction box and subjected to 4% isoflurane/air mixture (450 mL/min; Intervet, Sweden). Once the rat was anesthetised, the mixture was reduced to 2% and the rat was fixed in a stereotaxic frame (Kopf Instruments, Tujuga, CA, USA). The head of the rat was cleaned with chlorhexidine, and local anesthesia (Marcain, 2mg/kg, AstraZeneca) was applied subcutaneously before a longitudinal incision was made to reveal the skull. A burr hole 1 mm in diameter was made on the right side at coordinates AP (from skull Bregma, 5.3 mm) and ML (from skull midline, 4.5 mm).^62^ *Dura mater* was carefully removed using a sharp sterile needle tip. Subsequently, 0.4 μL pre-diluted KA solution (1 mg/mL) was injected via a glass capillary attached to a Hamilton syringe (Hamilton, Switzerland) at coordinates DV −3.2 relative to *dura mater* (Figure 2B). The KA was injected at a rate of 0.1 μL/15 s, and the capillary was left in place for 2 min after administration to prevent backflow. Immediately after capillary retraction, the wound was closed using staples, and the rats were given 1 mL saline (subcutaneously, 9 mg/mL).

### Treatment Individualization with MRI

The rats were placed in an anesthesia induction box containing 4% isoflurane. Once the rat was anaesthetized, the metal staples closing the incision were removed, and the animal was rapidly transferred to the MRI machine (Bruker 9.4T) and positioned on an animal holder inside an 86 mm volume radiofrequency (RF) coil. For optimal image quality, a 4 element rat brain surface RF coil was positioned on the head. After a quick imaging acquisition to verify the positioning of the animal, 22 slices with an in-plane resolution of 98 μm and a slice thickness of 0.5 mm were acquired to cover the hippocampal structure. T2 weighted images were obtained (4 averages, scan time: 6 min and 24 s) using a rapid imaging with refocused echoes (RARE) sequence (rare factor 8) with an effective echo time of 41 ms and a repetition time of 3,000 ms (Figure 2C). The image sets were analyzed with Osirix Lite software and aligned to a stereotaxic atlas.^62^ Aiming to administer the viral vector to the dorsal and ventral part of the hippocampus, ipsilateral to the KA injection, three injection points were defined. One ventral injection was targeted to the DG of the rostral hippocampus, and one injection with two vertically aligned depositing points targeted the caudal part of the dorsoventral hippocampal parenchyma. While defining the individualized coordinates, care was taken to account for any lesion resulting from the KA injection. Distortion of the hippocampal formation caused the injection coordinates to differ, highlighting the importance of MRI guidance following i.h. KA (Figure S2).

### EEG Video Monitoring

Implantation of the electrodes and transmitters for wireless video-EEG monitoring was done after the MRI session due to the incompatibility with the magnetic fields. The procedure was performed essentially as described previously.^39^ First, the rats were anesthetized with 4% isoflurane and placed in the stereotaxic frame. The transmitter (F40-EET, Data Sciences International, St. Paul, MN, USA) was placed in a subcutaneous pocket on the rats’ back. One stainless steel electrode (Plastics One, Roanoke, VA), soldered to the wire, was placed contralateral to the KA injection at the following coordinates: AP, −4.8; ML, −5.2 (left); DV, −6.2 (Figure 2B). The placement contralateral to the KA injection was chosen so as not to interfere with the viral vector injection. Also, previous unpublished observations from our group indicate that seizure activity propagates almost immediately from one hemisphere to the other, when recorded by two depth electrodes placed bilaterally in the hippocampus. Thus, the contralateral placement of the depth electrode should provide a good readout for the seizures in the kainate-injected hippocampal focus. The second electrode (just the lead wire) was placed on top of *dura mater* above the ipsilateral motor cortex. Both reference electrodes were placed on the *dura mater*, rostral to the lamboid structure. Two stainless screws were attached to the skull bone and the whole arrangement was covered by dental cement, leaving free the bregma and the area reserved for the viral vector administration (Figure 2D). To begin the video-EEG monitoring, the wireless transmitter was activated by a magnet and the cage was placed on top of a receiver unit (Data Sciences International, St. Paul, MN, USA). Four cameras (Axis, Lund, Sweden) were used to record the animal activity at all time. During the dark hours, infrared lamps were used to illuminate the setting. The data were collected and analyzed with A.R.T. and NeuroScore software (Data Sciences International, St. Paul, MN, USA) by an experimenter blind to the treatment identity of the animals. EEG was used as the primary element for detecting seizures, and the video aided confirmation of the convulsive seizures.

### Viral Vector Administration

The same vectors were used in the chronic efficacy study as in the dose-response study. However, the viral vectors were diluted to the working stock titer (10^12^ genomic particles/mL) in sterile PBS containing 1 mM MgSO_4_. The viral vector injections were essentially performed as described previously.^39^ The animals were anesthetized with 4% isoflurane (450 mL/min; Intervet, Sweden) and fixed in a stereotaxic frame (Kopf Instruments, Tujuga, CA, USA) before having their head shaved and cleaned with chlorhexidine. Local anesthetic Marcain (2mg/kg; AstraZeneca) was applied subcutaneously before the skull was exposed by a longitudinal incision. The injection site was located using the bregma as reference. Precaution was taken during the preceding implantation of the transmitter not to cover either the bregma or the vicinity of the skull bone around the planned viral vector injection with dental cement, thereby leaving these areas clear for the viral vector injection procedure (Figure 2D). A hole was drilled in the skull above the target region and *dura mater* was removed with the tip of a sterile needle. The injecting device consisting of a thin glass capillary attached to a Hamilton syringe was loaded with sterile water, an air bubble (1 μL), and the AAV vector suspension (1.2 μL). Subsequently, the glass capillary was lowered to the injection point in the brain. A total volume of 3 μL was infused into three locations with 1 μL viral vector suspension (10^12^ gp/mL, 0.2 μL/min) in each location (i.e., 1 μL in dorsal hippocampus rostrally and 2 μL in dorsal/ventral hippocampus caudally). The glass capillary was kept in place for 5 min after the injection before slowly retracting it to prevent backflow of the solution. Finally, the wound was cleaned up and closed with staples.

### Histology

After the final 3 weeks of video-EEG monitoring, the rats were deeply anaesthetized by pentobarbital (200 mg/kg, intraperitoneally) and subsequently decapitated. The brain was rapidly removed and placed on dry ice. 16 μm sections was with a cryostat and the samples were stored in -80°C until further processing. The sections were fixed in 4% paraformaldehyde for 20 min and then washed three times in Potassium PBS (KPBS) for 10 min and preincubated in 10% normal goat serum (NGS), 0.25% Triton X-100 in KPBS (T-KPBS) for 1 h. After adding rabbit anti-NPY antibody (1:500, #N9528, Sigma-Aldrich, Denmark) in 5% NGS, 0.25% Triton X-100 in KPBS sections were incubated overnight at 4°C. Sections were then washed three times for 10 min in T-KPBS, incubated for 2 h at room temperature (RT) with Alexa555Plus goat anti-rabbit antibody (1:500, Thermo-Fisher, USA) in 5% NGS and 0.25% Triton X-100 in KPBS, and washed once for 10 min in T-KPBS and twice for 10 min in KPBS. For NPY co-stainings, the slices were re-blocked in 10% NGS, T-KPBS at RT for 1 h and then incubated in either mouse anti-GFAP (1:500, #G3893 Sigma-Aldrich, Denmark) or mouse anti-NeuN (1:100, MAP377, Millipore) in 5% NGS, T-KPBS at RT for 2 h. The sections were then washed three times in T-KPBS for 10 min before the secondary antibody, Alexa488 goat anti mouse (1:200, Thermo-Fisher, USA), was applied in 5% NGS, T-KPBS for 2 h in RT. The sections were washed once in T-KPBS for 10 min and then twice in KPBS for 10 min.

The slides were then mounted with 1,4-diazabicyclo[2.2.2]octane (DABCO) (Sigma-Aldrich, DK) medium, and digitized images were obtained using Olympus BX61 microscope and CellSens software. Confocal images were obtained with a Nikon A1 microscope using a 60× objective with pixel size 0.05 μm.

Subsequently, optical density measurements were performed in Fiji (image processing software^63^) by an experimenter unaware of the treatment identity of the slices. The CA1, CA3, and molecular layer in the DG were examined by assigning an equally large rectangle in the ROI both ipsilateral and contralateral to the viral vector injection. The brightest part of the region was always selected for measurement. The optical density within these areas was then used to form a ratio between the hemispheres.

The procedure for Y2 functional binding was previously described in detail.^37^ Sections were rehydrated for 10 min at RT in buffer A (50 mM Tris-HCl, 3 mM MgCl, 0.2 mM ethylene glycol tetraacetic acid, 100 mM NaCl, pH 7.4) and then pre-incubated in buffer B (buffer A plus 0.2 mM dithiothreitol, 1 mM 1,3-dipropyl-8-cyclopentylxanthine, 0.5% w/v BSA, 2 mM guanosine-50-diphosphate [#G7127, Sigma-Aldrich], 0.1% dimethyl sulphoxide) for 20 min at RT. The sections were then incubated for 1 h at 25°C in buffer B with 40 pM [35S]-GTPγS (1250 Ci/mmol; NEG030H250UC; PerkinElmer, Denmark), 1 μM NPY (Schafer-N, Copenhagen, Denmark), 1 μM Y1 receptor antagonist BIBP3226 (#E3620, Bachem, Switzerland), and 10 μM Y5 receptor antagonist L-152,804 (#1382, Tocris Cookson, UK). To confirm Y2 receptor binding, 1 μM Y2 receptor antagonist BIIE0246 (#1700, Tocris Cookson, UK) was added to NPY together with BIBP3226 and L-152,804. Basal binding was determined by incubation without addition of NPY or antagonists, and non-specific binding was determined by incubation in buffer B with 40 pM [35S]-GTPγS and 10 mM non-labeled GTPγS (#89378; Sigma-Aldrich). The incubation was terminated by two 5 min-washings in ice-cold 50 mM Tris-HCl buffer (pH 7.4). Subsequently, the sections were dried and exposed to Kodak BioMax MR films together with 14C-microscales (Amersham Life Sciences) for approximately 5 days at 20°C. Finally, the films were developed in Kodak GBX developer.

### Statistics

In the cases where normal distributions could not be assumed, Mann-Whitney (two comparisons) or Kruskal-Wallis (three comparisons) was used for between-group comparisons. Distributions passing a normality test were compared with a Student’s t test (similar variance) or Welch test (not similar variance). In the case of multiple comparisons, the one-way ANOVA (one independent variable) or the two-way repeated-measures ANOVA (two independent variables, repeated-measures) were used with appropriate post hoc tests. The chi-square test was utilized to conclude if observed occurrence of a defined outcome in one group differed from the occurrence of the same outcome in another group. Correlations between variables were tested with the Pearson’s correlation test. Finally, the Kolmogorov-Smirnov test was used to compare non-normal distributions with a large sample size. Data are presented as mean ± SD. The level of significance was set at p < 0.05 except for the Kolmogorov-Smirnov tests with large sample sizes, where the significance level was adjusted to a more conservative p < 0.01 due to the high number of included values.^64^, ^65^

Gaussian curves were fitted to the inter-SRS interval data with the curve fitting package in MATLAB (Mathworks, MA, USA). The best Gaussian fit was achieved by three terms giving the formula:

$$a_{1}\times e^{-{\left(\frac{x-b_{1}}{c_{1}}\right)}^{2}}+a_{2}\times e^{-{\left(\frac{x-b_{2}}{c_{2}}\right)}^{2}}+a_{3}\times e^{-{\left(\frac{x-b_{3}}{c_{3}}\right)}^{2}},$$

where the terms represent distinguishable peaks in the distribution with center *b* and width *c*. The *b* and *c* values were used to extract the data from the distribution for further analysis. The values for the NPY/Y2 and empty groups’ inter-SRS interval distributions are presented in Table 1.

**Table 1:** Coefficients for the Best Three Term Gaussian Curve Fit of the Seizure Interval Data

	$a_{1}$	$b_{1}$	$c_{1}$	$a_{2}$	$b_{2}$	$c_{2}$	$a_{3}$	$b_{3}$	$c_{3}$
NPY/Y2	13.11	2.496	4.804	2.047	20.62	8.473	1.022	46.3	7.394
Empty	14.54	3.001	3.239	3.123	17.09	9.252	2.223	43.46	3.674

## Author Contributions

Conducted the experimental work: E.M., A.N., U.A.; Conceived and designed the study: E.M., C.R.G., D.P.D.W., M.K.; Analyzed the data: E.M., A.N., C.R.G., M.A., D.P.D.W., M.K.; Wrote the manuscript: E.M., C.R.G., D.P.D.W., M.K.

## Conflicts of Interest

This study was sponsored by the company CombiGene AB. M.K. and D.W. are co-founders and consultants of this company.

## Supplemental Information

Document S1. Figures S1–S6

Document S2. Article plus Supplemental Inforomation
