# Supplementary material for: Disease Modification by Combinatorial Single Vector Gene Therapy: A Preclinical Translational Study in Epilepsy
Source: Mol Ther Methods Clin Dev. 2019 Sep 18;15:179–93. doi: 10.1016/j.omtm.2019.09.004 (PMC6807261; doi:10.1016/j.omtm.2019.09.004)
Supplement: Document S1. Figures S1–S6 [file mmc1.pdf]

OMTM, Volume *15*

Supplemental Information

Disease Modification by Combinatorial Single Vector Gene Therapy: A Preclinical Translational Study in Epilepsy

Esbjörn Melin, Avtandil Nanobashvili, Una Avdic, Casper R. Gøtzsche, My Andersson, David P.D. Woldbye, and Merab Kokaia

Supplementary information

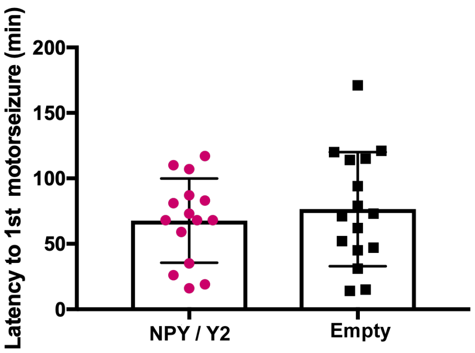

Supplementary figure S1. The epileptogenic insult, in terms of latency to 1st motor seizure, did not differ between the treatment groups (Unpaired t-test, p = 0.53, n_{NPY} = 15, n_{Empty} = 16).

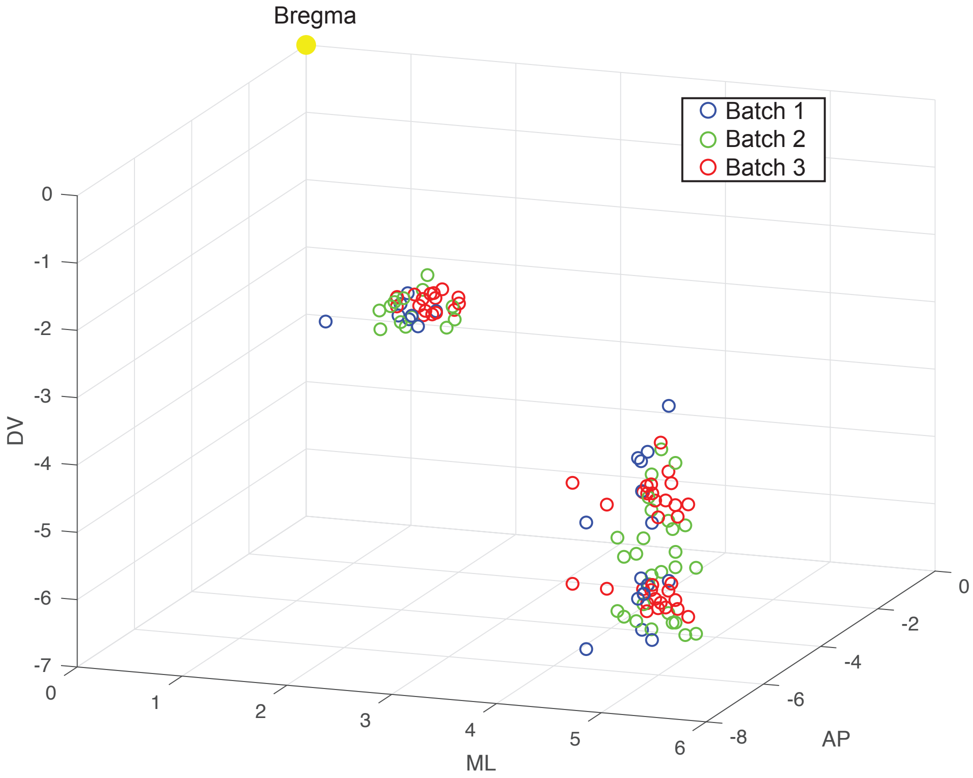

Supplementary figure S2. The injection coordinates were individualized for each rat by MRI. The mean dorsal coordinate was: AP: -4.07 ± 0.26 mm, ML: 2.17 ± 0.27 mm, DV: -2.46 ± 0.16 mm. The mean ventral coordinates were: AP: -5.71 ± 0.40 mm, ML: 4.86 ± 0.20 mm, DV1: -4.53 ± 0.54 mm, DV2: -6.05 ± 0.30 mm.

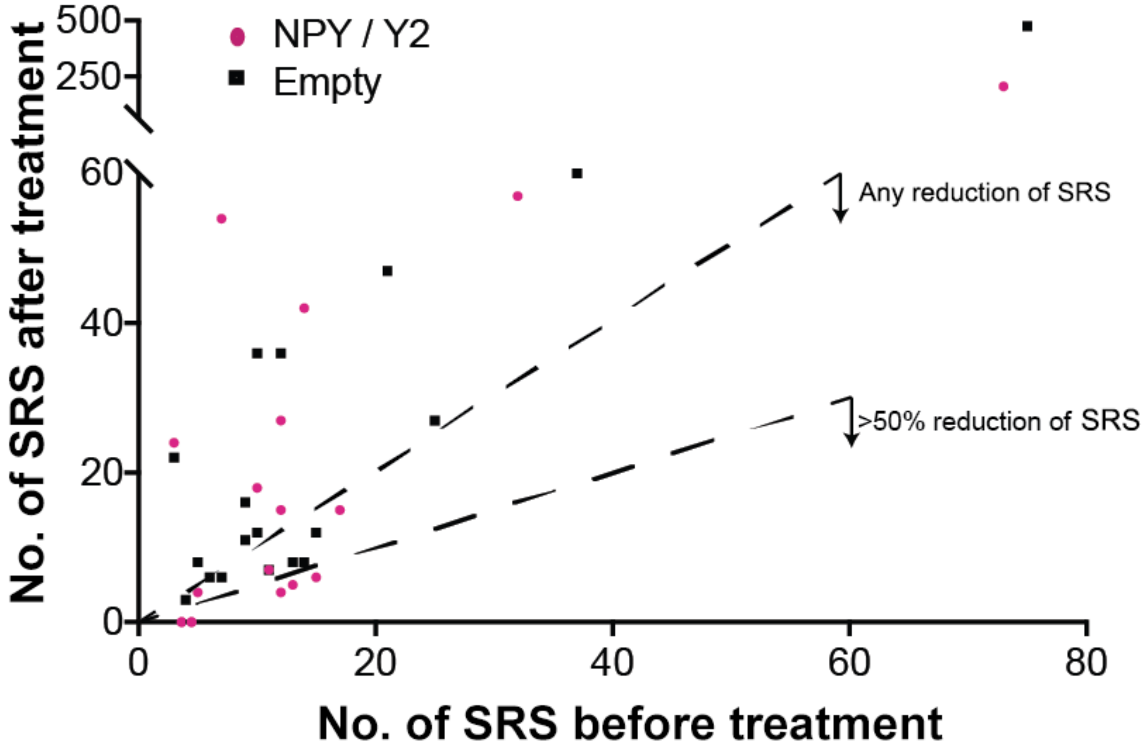

Supplementary figure S3. The variance in the number of SRS both before and after treatment was large in animals treated with either NPY/Y2 or control. Also, animals with a decreased SRS frequency after treatment/sham were represented in both groups. Rats located below the dashed lines display any level of decreased SRS frequency (upper) or more than 50% decrease in SRS frequency (lower).

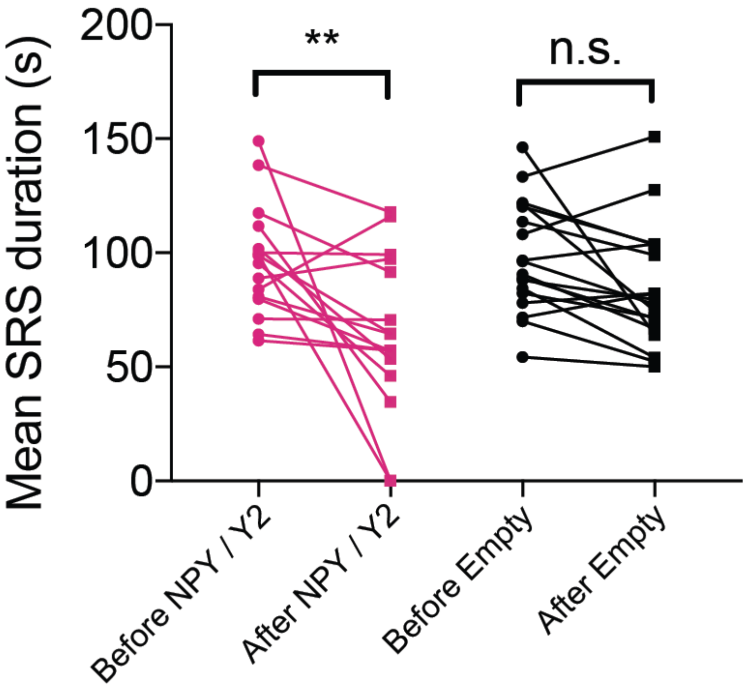

Supplementary figure S4: The mean SRS duration was measured before and after viral vector administration. The NPY/Y2 treatment resulted in a significant reduction of the mean SRS duration. No difference was found when comparing the pre- and post-treatment values in the control (Empty) group. (Holm-Sidak's Post-hoc test following significant one-way ANOVA, $^{**}p < 0.01$, $n_{NPY} = 16$, $n_{Empty} = 18$).

NPY / Y2 - Pre treatment

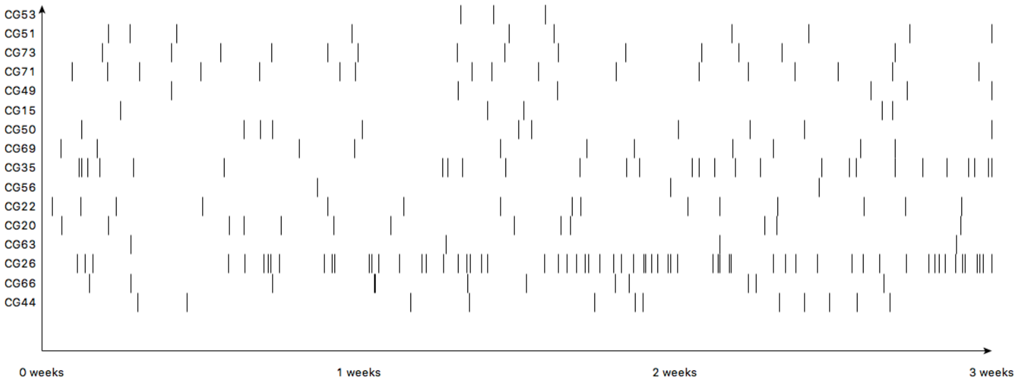

NPY / Y2 – Post treatment

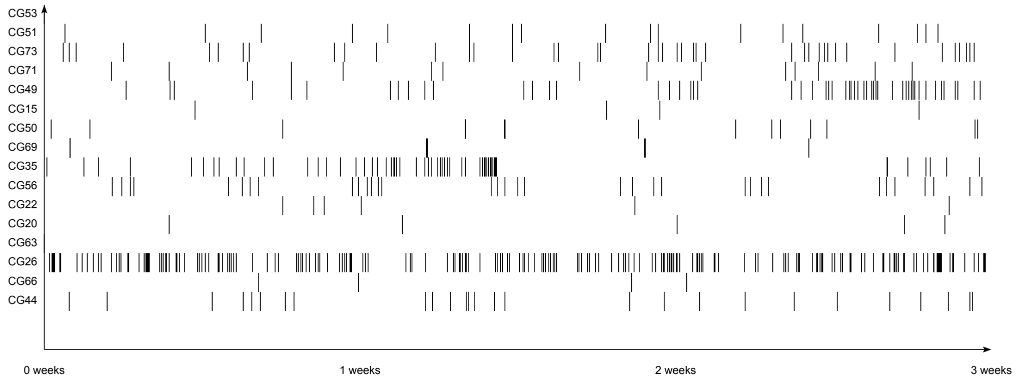

Empty – Pre treatment

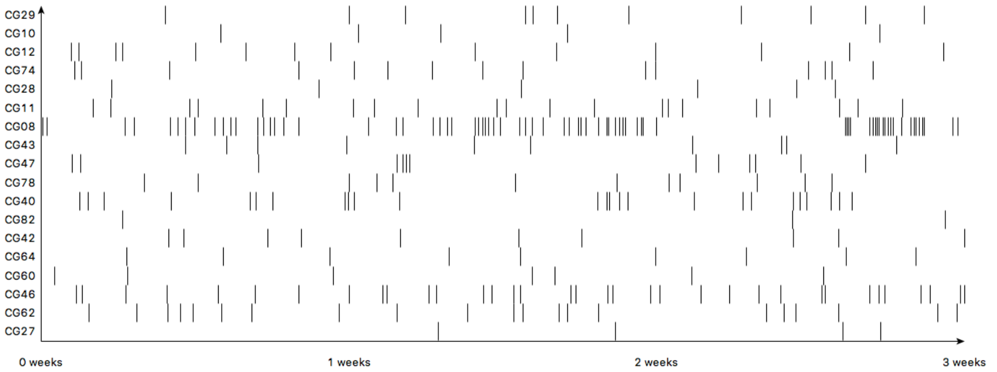

Empty – Post treatment

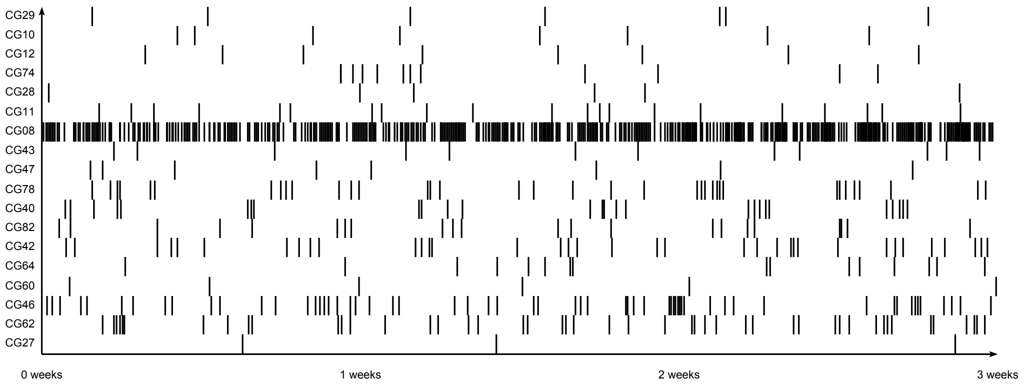

Supplementary figure S5. The full dataset of SRS plotted as they occurred during the pre- and post-treatment monitoring period. Clustering can be found in both groups.

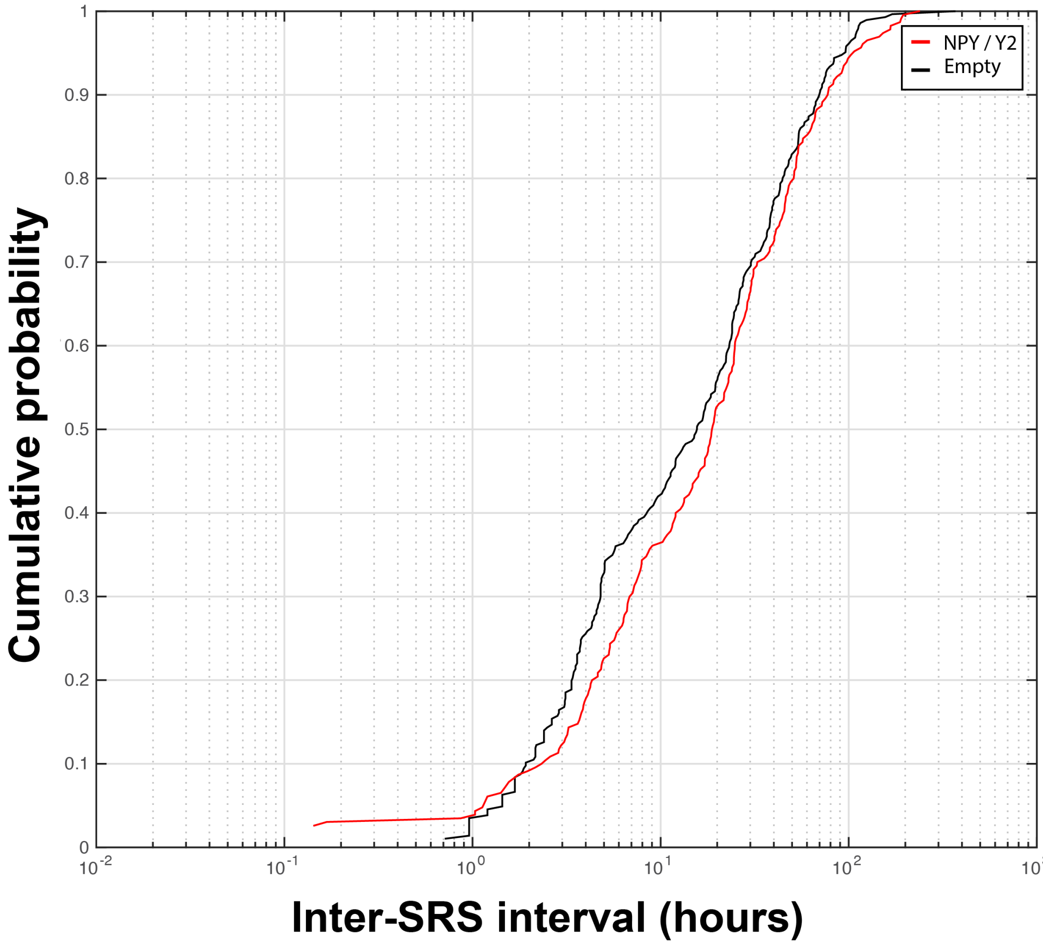

Supplementary figure S6. No difference was found in the distribution of inter-SRS intervals before intervention (Kolmogorov-Smirnov, $p = 0.047$), indicating successful stratification of the groups.
